# Statistical assessment of natural radioactivity, radon activity, and associated radiological exposure due to artisanal mining in Atiwa West district of the Eastern region, Ghana

**DOI:** 10.1016/j.heliyon.2024.e34705

**Published:** 2024-07-17

**Authors:** Esther Osei Akuo-ko, Francis Otoo, Eric Tetteh Glover, Eunice Amponsem, Amin Shahrokhi, Anita Csordás, Tibor Kovács

**Affiliations:** a Institute of Radiochemistry and Radioecology, University of Pannonia, Hungary; b Radiation Protection Institute, Ghana Atomic Energy Commission, Ghana; c GIS Department, Wallulel Ghana Limited, Ghana

**Keywords:** Natural radionuclides, Exhalation rates, Rn-222, CR-39 detector, Radiation dose

## Abstract

The activity concentration of natural radionuclides, radon activity concentration, mass and area exhalation rates have been studied in soils from gold mining communities in Atiwa West district. The natural radionuclides were determined by gamma ray spectrometry method while radon concentrations were measured using CR-39 detectors. The mean activity concentrations were found to be 26.9 ± 1.7 Bq/kg, 57.5 ± 3.6 Bq/kg, 237.5 ± 17.6 Bq/kg and 560.0 ± 54 Bq/m^3^ for Ra-226, Th-232, K-40 and Rn-222 respectively. The evaluated mass exhalation rates ranged from 2.8 ± 0.3 to 6.5 ± 0.7 × 10^−5^ Bq/kg/h while the area exhalation rates were from 0.8 ± 0.09 to 2.0 ± 0.21 × 10^−3^ Bq/m^2^/h. Some mining and farming areas recorded high exhalation rates indicating that the use of soils as building materials from such areas could pose a level of radiation hazard to the population. The evaluated radiological risks were below reference levels. A good linear correlation was observed between Ra-226 and Rn-222 activity concentrations and in the investigated soils. The Pearson correlation coefficient, cluster analysis and principal component analysis were used to study the relationship between the determined parameters of the study.

## 1 Introduction

All soils and rocks contain natural radionuclides at varying concentrations depending on type and geological location [1]. Soil serves as a medium of migration for radionuclides transfer to biological systems, a primary source of continuous radiation exposure to humans and a basic indicator of radiological pollution in the environment. Furthermore, soil radioactivity is usually significant for creating baseline data for future radiation impact assessment and radiation protection [2,3]. The external exposure to radioactivity levels is mainly caused by gamma radiations from U-238 (or Ra-226), Th-232 and K-40 in soils and rocks as well as building materials while internal exposure is caused by alpha and beta radiations from the radioactive radon gas [1,4]. Radon is mainly emitted from rocks and soil. Building materials are also potential sources of radon in indoor environments [5].

Radon, which is a radioactive noble gas occurs in the environment as Rn-222 (radon), Rn-220 (thoron) and Rn-219 (actinon) isotopes from the decay series of U-238, Th-232 and U-235, respectively [6]. The Rn-222 isotope (hereinafter known as radon) is the most considered for radiological safety since it has long half-life of 3.8 days and able to accumulate in the atmosphere after exhalation. The inhalation of its radioactive decay products, Po-218, Po-214, Pb-214 and Bi-214, is the radiological risk associated to radon [6,7]. The decay of Ra-226 (from U-238 series) leads to the release of radon gas in indoor and outdoor environment, soil, water, ground and subsequently the increase in airborne radon concentration [8].

Numerous epidemiological research, as well as biophysical modelling analyses, have verified the damage to human health caused by radon gas accumulation in indoor and outdoor areas. All potential mitigation strategies are focused on monitoring radon concentrations in workplaces and homes [9]. In recent years, international bodies concerned in radiation protection and public health have produced a standardized framework for ionizing radiation protection as well as criteria aimed at greater protection from natural radiation exposure [10]. An important objective of the Council Directive 2013/59/Euratom of the EU basic Safety Standards, is the protection against long-term risks associated with the exposure to radon in dwellings and at workplaces [11,12].

Radon in homes and workplaces poses a major health risk above a particular level of radon concentration as there is evidence of correlation between radon and health risk [13,14]. The greatest health risk linked with the long-term elevated radon exposure is an increased risk of developing lung cancer, which varies according to radon concentration and duration of exposure. Hence, radon has been recognized as the second cause of lung cancer by the World Health Organization (WHO) [15,16]. Approximately 15 % of the lung cancer cases globally has been recognized to be caused by radon exposure [17] and responsible for 3%–14 % of all lung cancer deaths [[18], [19], [20]]. Radon concentrations can also lead to toxicity in the blood and bone marrow [21]. The long-term exposure to high radon levels also leads to pathological consequences such as respiratory functional changes because of internal exposure of the respiratory tract to the alpha particles and therefore has been classified as occupational respiratory carcinogen [22,23]. It has been estimated that radon and its decay products account for about 50 % (1.15 mSv/y of the total 2.4 mSv/y) of the radiation dose received by the public [5,6,24]. The concentrations of indoor and outdoor radon differ from location to location depending on geological features and meteorological conditions of the location [15].

Radon exhalation rate describes the escape of radon from rock and soil surfaces into the atmosphere. It is the emission of radon per unit area per unit time [[24], [25], [26]]. During this process radon atoms, produced because of Ra-226 disintegration in the soil grains, escape by moving through the soil pores until it reaches the surface and are released into the environment [27,28]. Radon exhalation is affected by several factors such as the chemical and physical characteristics of the soil, Ra-226 content, permeability, soil humidity, soil structure, emanation coefficient, changes in meteorological factors [24,29,30]. Soil is the ultimate source of radium and radon [31], and because radon is formed from Ra-226 via alpha decay, the radium concentration of a sample also influences the level of environmental radon. Higher soil Ra-226 values contribute greatly to the increase of environmental radon [2]. Soil radon exhalation rate is a key parameter for assessing the radon level in the local environment and its radiological hazard [25,28].

Mining is an important anthropogenic activity which leads to increase in soil radon exhalation rates. Thus, mining can redistribute and enhance the activity concentrations of natural radionuclides including radon in the environment and subsequently their accumulation in the environment [27]. Unregulated mining such as artisanal and small-scale mining activities occurring in the study area could lead to severe human and environmental harm as radionuclides in dust, soil, and water when inhaled or ingested result in human health implications such as cancers and respiratory diseases [[22], [23], [24]]. In Ghana, mining has neither been subjected to radiological control nor has there been enough studies on the radiological effects of mining at the various mining sites and towns and their immediate environments including residential, nonresidential, and farming areas. As a result, it is necessary to determine the radioactivity concentrations and radon exhalation rates in soils for correct evaluation of the potential radiological risks of mining to humans [25]. Soil radon exhalation rate has widely been studied [19,20,[32], [33], [34], [35], [36], [37], [38], [39], [40], [41], [42], [43]] worldwide. However, data on soil radon exhalation rate in Ghana is scarce, with previous studies limited to specific areas [[44], [45], [46], [47]]. Since such studies has not yet been carried out at the present study area, it is important to undertake this investigation to obtain data on radioactivity levels and to evaluate the radiation risks resulting from soils in the Atiwa West mining areas as well as their use for building construction. Therefore, purpose of the present study is to determine the natural radioactivity levels, radon activity concentration, radon exhalation rates (mass and area exhalation) in soils of mining areas within Atiwa West of Eastern region, Ghana and to assess the health hazards due to radioactivity among the populace of the study area.

## 2 Materials and methods

### 2.1 Description of study area

The study area is in the Atiwa West district of the Eastern region of Ghana. The district capital, Kwabeng and other major towns have several artisanal mining activities on-going in the district. The area is located in Ghana's semi-deciduous forest area, which consists of tall trees with evergreen undergrowth. The soils are composed of sandy loam topsoil with underlying gritty clay loam and clay subsoil containing high concentrations of quartz gravel, stones, and ironstone. Soil is used as a building material in some of the dwellings in the area [45]. The landscape is gentle and undulating (240 m–750 m above sea level) with different rocks formation resulting in its different relief features, ranging from flat bottom valleys to steep-sided high lands which are covered with iron pans, gold, bauxite, and kaolin and with the steep-sided highlands creating some waterfalls [[48], [49], [50]].

### 2.2 Sample collection and preparation

**Fig. 1:**
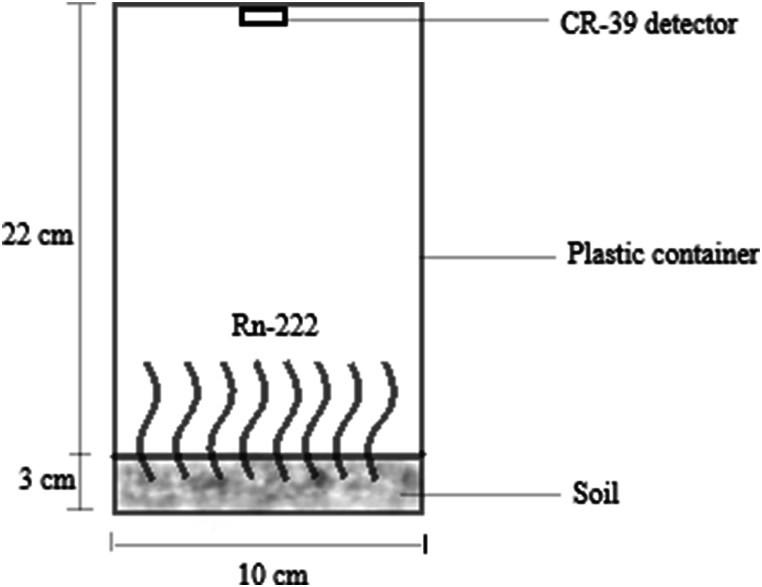
Experimental setup for radon exhalation rate measurement.

Soil samples were collected from 22 different locations of mining, farming and undisturbed (residential and nonresidential) areas in the Atiwa West district. At each location, soil samples were collected from 2 to 3 sampling points into well labelled Ziploc bags. A total of fifty-six sampling sites were sampled for analysis. Soil samples were collected from different locations with each sampling site covering a surface area of 2 m × 2 m. Surface soils were collected with a uniform sampling depth of 10–30 cm for all sampling locations. Soil samples were collected in pairs, one for radon exhalation measurement and the other for gamma ray spectrometry analysis. In the laboratory, all samples were air-dried for about a week and oven dried at 60 °C for 24 h. Soil samples for gamma ray spectrometry were pulverized, homogenized, and sieved to a consistent particle size of 5 μm. They were weighed into labelled Marinelli beakers and sealed. The sealed samples were kept at room temperature for 4 weeks to allow the Ra-226 and Rn-222 decay series to achieve radioactive equilibrium with the short-lived progenies while also preventing radon gas escape. 300 g of the soil samples for radon exhalation measurements were weighed into cylindrical vessels of 25 cm height and 10 cm diameter with CR-39 detector at the top as shown in Fig. 1. The samples were for 90 days to detect radon gas.

### 2.3 Activity concentration of radionuclides

The activity concentrations of Ra-226, Th-232 and K-40 I soils were estimated using energy lines of the progenies; Bi-214 (609.31 keV, Pγ: 45.2 %) and Pb-214 (351.93 keV, Pγ: 35.3 %) representing the activity of Ra-226. To determine the activity concentration of Th-232, energy lines of Tl-208 (583.2 keV, Pγ: 30.5 %; 2614.5 keV, Pγ: = 35.8 %) and Ac-228 (911.20, Pγ: = 26 %; 968.97 keV, Pγ: 16 %) were employed. The activity concentration of the Ra-226 and Th-232 was determined using the average peak for nuclide with more than one offspring. The K-40 concentration was determined using only the 1460.82 keV (Pγ: = 10.6 %) peak line. The background concentration was determined using an empty Marinelli beaker with the same geometry as the measured sample before the soil samples were counted with High Purity Germanium (HPGe) gamma detector. Multiple sealed sources of Am-241, Co-60 and Cs-137 were used for detector calibration to ensure precise peak identification. The counting time was done at 72,000 s for better gamma statistics and minimal counting error. The Genie 2000 V3.3 spectroscopic software from Canberra was used for data capture, presentation, and online spectrum analysis [44,51]. Using the following analytical equation, the activity concentration (A) (Bq/kg) of each radionuclide in any given sample was derived from the spectrum.

(1) $$A=\frac{N}{P\left(E\right)*\eta \left(E\right)*T*M}$$

where, A is the activity concentration of Ra-226, Th-232 and K-40 in Bq/kg, *N* is the net peak area, *P(E)* is the probability of gamma emission, η*(E)* is the photo peak efficiency resulting from the standard, *T* is the measurement time in seconds and *M* is the mass of the sample in kg [18,45].

### 2.4 Radon exhalation measurement

The radon exhalation rate in soil samples was determined using the passive Rn-222 CR-39 Solid State Nuclear Track Detector (SSNTD). The detectors were acquired from Radosys (Hungary) and traceable to the laboratory for radon calibration at the Federal Office for Radiation Protection (Germany). The determination of the exhalation rate was done with a tightly closed vessel method with a cylindrical jar. To achieve equilibrium between radium and radon, a known weight of 300 g of each sample was placed at the bottom of the cylindrical vessel and totally sealed for one month. To count for only Rn-222 and prevent Rn-220 from evading the surface, a CR-39 detector was installed at the top of the chamber covering at a distance of 22 cm from the surface of the sample. Rn-220 has a half-life of 15 s and hence placing soil samples very low at the bottom of the chamber extends the distance (22 cm) the radionuclide must travel before reaching the surface of the CR-39 detector of which by then it would have decayed. As presented in Fig. 1, the soil samples were placed at the bottom of the cylindrical vessel with 25 cm height and 10 cm diameter for 90 days to detect radon exhalation [[43], [44], [45], [46]].

### 2.5 Etching and determination of radon concentration

Following the exposure, the detectors were removed and etched in a 6.25 N solution (1 L of distilled water and 1 kg of NaOH) at 90 °C for 5 h at constant temperature, then the detectors were neutralized in 36 ml of 96 % diluted acetic acid for 15 min. Finally, the detectors were rinsed in distilled water for another 15 min to remove any excess chemicals and dried for 4 days. Using an optical microscope with a 40× magnification objective lens, the latent tracks generated on the detectors were scanned with RadoMeter and tallied in 144 fields. The density of the tracks left on the track films was then utilized to calculate the radon concentration [46]. To estimate radon parameters, the following mathematical expressions were used.

#### 2.5.1 Radon concentration

(2) $$C_{\text{Rn}}=\frac{\varrho }{\varepsilon *t}$$

Where, C_Rn_ is the activity of radon concentration in Bq/m^3^, **ρ** is the measured track surface density in track/cm^2^, ***ε*** is the detector calibration factor in track/cm^2^d/(Bq/m^3^) and t is the exposure time in s.

#### 2.5.2 Radon exhalation rate

The radon exhalation rates in terms of area (E_A_) and mass (E_M_) were evaluated with the relations below:

(3) $$E_{A}=\frac{CV\lambda }{A\left[\left(T+1/\lambda (e⌃(-\lambda T\right)-1)\right]}$$

(4) $$E_{M}=\frac{CV\lambda }{M\left[\left(T+1/\lambda (e⌃(-\lambda T\right)-1)\right]}$$

where, E_A_ is the area radon exhalation rate in mBq/m^2/^h, E_M_ is the mass radon exhalation rate in mBq/kg/h, C is the equilibrium radon activity in Bq/m^3^, V is the volume of the diffusion chamber in m^3^, A is the area of the diffusion chamber in m^2^, M is the mass of the sample in kg, ***λ*** is the decay constant in hours and T is the exposure time in hours [36,38,42].

### 2.6 Radiological hazards assessment

#### 2.6.1 Radium equivalent activity

The radium equivalent activity (Ra_eq_) was determined using the expression below, given that 370 Bq/kg of Ra-226, 259 Bq/kg of Th-232 and 4810 Bq/kg of K-40 produce the same gamma dose rate:

(5) $${\text{Ra}}_{\text{eq}}=A_{\text{Ra}}+1.43A_{\text{Th}}+0.077A_{K}$$

where, A_Ra_, A_Th_ and A_K_ are the activity concentrations of Ra-226, Th-232 and K-40, respectively in Bq/kg. The maximum allowed Raeq activity is 370 Bq/kg, which equates to an effective dosage of 1mSv/y [[52], [53], [54]].

#### 2.6.2 External hazard index

The external hazard index is used to assess the external radiological hazard posed by gamma rays from natural radioactive sources. The external hazard index (H_ex_) is supposed to equate to the radium equivalent's maximum allowable level of 370 Bq/kg. To achieve an annual effective dose of 1mSv/y, H_ex_ must be less than or equal to 1. The H_ex_ it is calculated by the equation:

(6) $$H_{\text{ex}}=\left(A_{\text{Ra}}/\;370\right)+\left(A_{\text{Th}}/\;259\right)+\left(A_{K}/\;4810\right)$$

where, A_Ra_, A_Th_ and A_K_ are the activity concentrations of Ra-226, Th-232 and K-40, respectively in Bq/kg [51,55].

#### 2.6.3 Internal hazard index

In order to achieve negligible dangerous effects of radon and its short-lived progeny on the respiratory organs, the internal hazard index (H_in_) values must be less than unity. This is calculated by the expression:

(7) $$H_{\text{in}}=\left(A_{\text{Ra}}/\;185\right)+\left(A_{\text{Th}}/\;259\right)+\left(A_{K}/\;4810\right)$$

where, A_Ra_, A_Th_ and A_K_ are the activity concentrations of Ra-226, Th-232 and K-40, respectively in Bq/kg [51,55].

#### 2.6.4 Absorbed gamma dose rate

The absorbed dose rates (D) from gamma radiations are used to compute doses in air at 1 m above ground for the uniform distribution of radionuclides Ra-226, Th-232, and K-40 in soil. It may be computed using known activity concentrations of Ra-226, Th-232, and K-40 and conversion factors of 0.462 nGy/h for Ra-226, 0.604 nGy/h for Th-232 and 0.0417 nGy/h for K-40. The absorbed dose rate was determined by the equation below:

(8) $$D=0.462A_{\text{Ra}}+0.604A_{\text{Th}}+0.0417A_{K}$$

Where, A_Ra_, A_Th_ and A_K_ are the activity concentrations of Ra-226, Th-232 and K-40, respectively in Bq/kg [54,56].

## 3 Results and discussion

### 3.1 Distribution of radionuclides

**Fig. 2:**
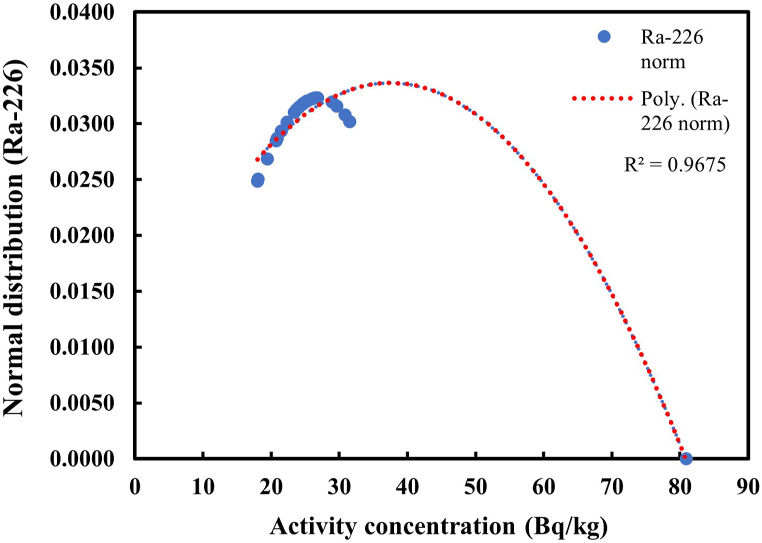
Distribution of Ra-226 in soil samples.

**Fig. 3:**
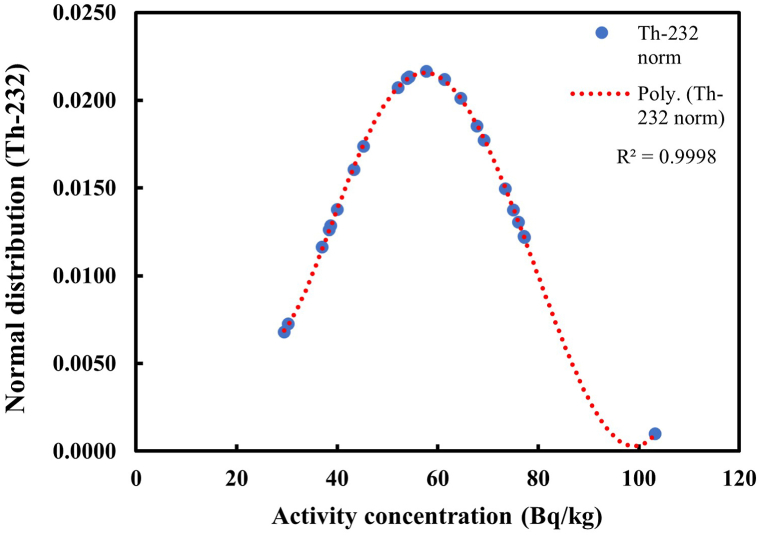
Distribution of Th-232 in soil samples.

**Fig. 4:**
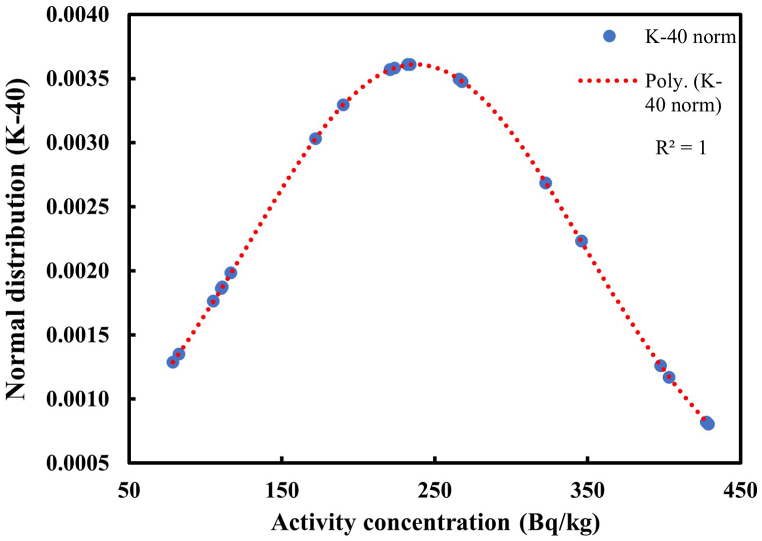
Distribution of K-40 in soil samples.

The frequency distribution of radionuclides in soil samples within studied area were done by plotting the normal distribution and the standard deviation of the measured activity concentrations of Ra-226, Th-232 and K-40 as shown in Fig. 2, Fig. 3, Fig. 4. The activity concentrations were determined in soils sampled from mining areas, farming areas and undisturbed areas (residential and non-residential) within the study area. The distribution of Ra-226 in soils of the study area showed a lack of normal distribution of the radionuclide with *p* values < 0.05 for both Kolmogorov-Smirnov and Shapiro-Wilk normality tests while Th-232 and K-40 showed normal distributions of data with p values > 0.05 for both normality tests. The lack of a normal distribution of data for Ra-226 can be due to the outlier value of 80.9 Bq/kg observed in a farming area (SL08) and the differences in the geological properties of the various sampling locations [57].

**Fig. 5:**
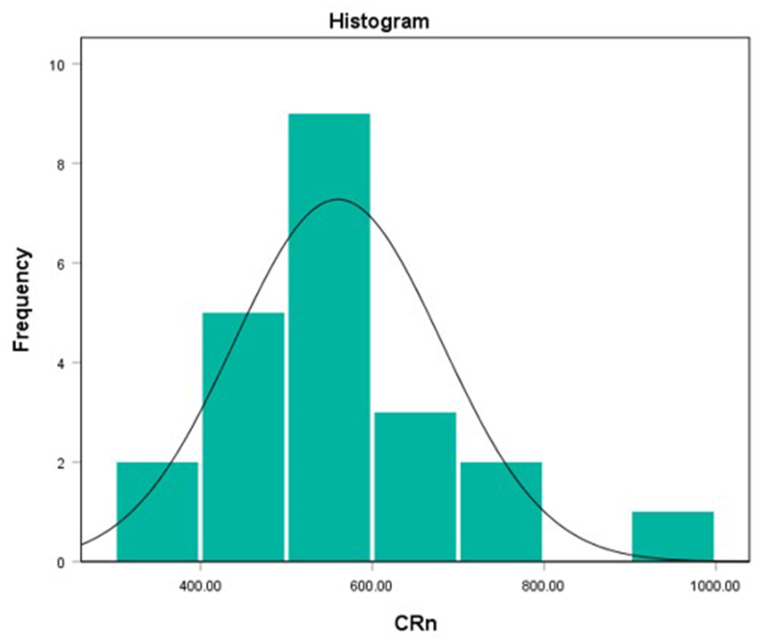
Frequency distribution of radon concentrations (CRn) in Bq/m.^3^.

The frequency distribution of the mean Rn-222 concentration for the sampling locations have been presented in Fig. 5. The Rn-222 data showed a normal distribution of radionuclides in the study area. The data had 0.20 and 0.13 for Kolmogorov-Smirnov and Shapiro-Wilk normality tests, respectively. The reason for the normal distribution of the radon data is the similarity in the lithologies in the study area.

### 3.2 Activity concentrations of radionuclides in different locations of the study area

The activity concentration of natural radionuclides measured in soil samples from the study area are presented in Table 1.

**Table 1:** Activity concentrations of Ra-226, Th-232 and K-40 measured in soil samples.

	Mining areas		Farming areas		Undisturbed areas	
	Range	Mean	Range	Mean	Range	Mean
**Ra-226 (Bq/kg)**	18.0 ± 1.2–26.4 ± 2.0	22.7 ± 1.6	19.4 ± 1.3–80.9 ± 3.2	36.5 ± 2.2	21.5 ± 1.2–30.8 ± 1.9	26.8 ± 1.6
**Th-232 (Bq/kg)**	30.3 ± 1.6–77.3 ± 5.1	63.4 ± 4.0	37.0 ± 1.7–103.3 ± 5.3	61.5 ± 3.4	29.5 ± 2.3–57.5 ± 3.8	43.6 ± 3.0
**K-40 (Bq/kg)**	78.8 ± 11.8–427.7 ± 21.6	221.5 ± 16.4	116.7 ± 10.2–429.1 ± 23.4	266.3 ± 18.9	110.5 ± 11.7–403.4 ± 28.0	243.0 ± 18.7

From Table 1, the activity concentrations of Ra-226 ranged between 18.0 ± 1.2 to 80.9 ± 1.4 Bq/kg with an average of 26.9 ± 1.7 Bq/kg. Th-232 activities were within the range 29.5 ± 2.8 and 103.3 ± 5.2 Bq/kg having a mean value of 57.5 ± 3.6 Bq/kg while the activities for K-40 were between 78.8 ± 17.1 and 429.1 ± 23.4 Bq/kg with a mean of 237.5 ± 17.6 Bq/kg. The radionuclides activity concentrations determined in the investigations revealed that Th-232 exist in abundance as compared to Ra-226 in soils and rocks of the study area. All sampling locations had Ra-226 concentrations below worldwide average of 35 Bq/kg except for SL08 (80.9 ± 1.4 Bq/kg) whereas Th-232 recorded values above worldwide average value of 30 Bq/kg for all sampling locations except location SL18 (29.5 ± 2.8 Bq/kg). For K-40, only 3 locations (SL08, SL15 and SL18) had concentrations above the worldwide average of 400 Bq/kg [54]. Activity concentrations of Ra-226 determined in farming areas were higher than the concentrations determined at mining sites and undisturbed lands. Average Ra-226 activity concentrations for mining, farming and undisturbed lands were 22.7 ± 1.6 Bq/kg, 36.5 ± 2.2 Bq/kg and 26.8 ± 1.6, respectively. The mean Ra-226 concentration was above worldwide average due to the highest Ra-226 concentration of 80.9 ± 1.4 Bq/kg determined at location SL08. For Th-232 activity concentrations in the study area, mining sites recorded the highest mean concentration of 63.4 ± 4.0 Bq/kg, followed by farming and undisturbed areas with 61.5 ± 3.4 Bq/kg and 43.6 ± 3.0 Bq/kg, respectively. K-40 mean activity concentrations were high in soils of farming areas (266.3 ± 18.9 Bq/kg) than in undisturbed (243.0 ± 18.7 Bq/kg) and mining areas (221.5 ± 16.4 Bq/kg).

The Rn-222 activity concentrations determined in the soil samples as shown in Table 2 were found to vary from 390.6 ± 38 to 907.5 ± 93 Bq/m^3^ with an arithmetic mean, geometric mean, and standard deviation of 560.0 ± 54.3 Bq/m^3^, 548.5 and 120.6, respectively. Rn-222 concentrations in soils from mining areas ranged from 390.6 ± 38 to 589.8 ± 54 Bq/m^3^, for undisturbed areas the concentrations varied between 472.2 ± 45 and 707.3 ± 71 Bq/m^3^ whereas farming areas had Rn-222 concentrations ranging from 415.8 ± 40 to 907.5 ± 93 Bq/m^3^. Mean Rn-222 concentrations were 503.5 ± 46.9 Bq/m^3^ for mining areas, 632.8 ± 63.4 Bq/m^3^ for farming areas and 602.8 ± 60.3 Bq/m^3^ for undisturbed areas. In general, undisturbed and farming areas recorded high radon activity concentrations (∼410 Bq/m^3^ and above) as compared to mining areas which recorded low concentrations (∼580 Bq/m^3^ and below). This could be attributed to the fact that at mining sites the soil is constantly being disturbed hence soils become loosed and less compact making it easy for radon gas to escape from soil pores into the atmosphere [24,[27], [28], [29]].

**Table 2:** Average Ra-226 and Rn-222 activity concentration, mass and area exhalation rates for the various sampling locations.

Location	Description of location	Ra-226 (Bq/kg)	CRn (Bq/m^3^)	E_M_ × 10^−5^ (Bq/kg/h)	E_A_ × 10^−3^ (Bq/m^2^/h)
SL01	Mining area	22.3 ± 1.8	497.4 ± 41	3.6 ± 0.3	1.1 ± 0.09
SL02	Mining area	18.0 ± 1.2	395.2 ± 32	2.8 ± 0.2	0.9 ± 0.07
SL03	Mining area	24.5 ± 1.4	544.2 ± 52	3.9 ± 0.4	1.2 ± 0.11
SL04	Mining area	24.1 ± 2.0	547.6 ± 50	3.9 ± 0.3	1.2 ± 0.08
SL05	Mining area	25.3 ± 1.8	563.4 ± 51	4.1 ± 0.4	1.2 ± 0.12
SL06	Farming area	26.8 ± 3.2	603.4 ± 62	4.3 ± 0.4	1.3 ± 0.13
SL07	Farming area	19.4 ± 2.4	415.8 ± 40	3.0 ± 0.3	0.9 ± 0.08
SL08	Farming area	80.9 ± 1.4	907.5 ± 93	6.5 ± 0.7	2.0 ± 0.21
SL09	Farming area	23.7 ± 2.6	525.0 ± 51	3.8 ± 0.4	1.1 ± 0.08
SL10	Farming area	31.5 ± 1.3	712.1 ± 71	5.1 ± 0.5	1.5 ± 0.11
SL11	Mining area	26.4 ± 1.4	589.8 ± 54	4.2 ± 0.4	1.3 ± 0.09
SL12	Mining area	26.0 ± 2.0	587.2 ± 55	4.2 ± 0.4	1.3 ± 0.11
SL13	Mining area	20.9 ± 1.2	455.8 ± 46	3.3 ± 0.3	1.0 ± 0.11
SL14	Mining area	23.4 ± 1.5	514.8 ± 51	3.7 ± 0.4	1.1 ± 0.10
SL15	Mining area	18.1 ± 1.6	390.6 ± 38	2.8 ± 0.3	0.8 ± 0.09
SL16	Mining area	20.7 ± 1.5	453.0 ± 46	3.3 ± 0.3	1.0 ± 0.09
SL17	Undisturbed area	28.9 ± 1.8	654.8 ± 66	4.7 ± 0.5	1.4 ± 0.12
SL18	Undisturbed area	24.7 ± 1.2	549.0 ± 58	3.9 ± 0.4	1.2 ± 0.08
SL19	Undisturbed area	29.6 ± 1.9	674.6 ± 64	4.9 ± 0.5	1.5 ± 0.12
SL20	Undisturbed area	21.5 ± 1.7	472.2 ± 45	3.4 ± 0.3	1.0 ± 0.08
SL21	Undisturbed area	25.1 ± 1.4	558.6 ± 58	4.0 ± 0.4	1.2 ± 0.14
SL22	Undisturbed area	30.8 ± 1.6	707.3 ± 71	5.1 ± 0.5	1.5 ± 0.14
**Average**		**26.9** ± 1.7	**560.0 ± 54**	**4.0 ± 0.4**	**1.2 ± 0.11**
Minimum		18.0 ± 1.2	390.6 ± 38	2.8 ± 0.3	0.8 ± 0.09
Maximum		80.9 ± 1.4	907.5 ± 93	6.5 ± 0.7	2.0 ± 0.21
Geomean		25.4	548.5	3.9	1.2
Standard deviation		12.6	120.6	0.8	0.3

± means standard error.

### 3.3 Evaluation of exhalation rates in different locations of the study area

The determination of Rn-222 mass and area exhalation rates of soil samples collected from the study were performed using a tightly closed vessel with a CR-39 detector and the results are presented in Table 2.

From Table 2, the Rn-222 mass exhalation rates of the soil samples were between 2.8 ± 0.3 × 10^−5^ and 6.5 ± 0.7 × 10^−5^ Bq/kg/h with an average of 4.0 ± 0.4 × 10^−5^ Bq/kg/h. The Rn-222 area exhalation rates ranged between 0.8 ± 0.09 × 10^−3^ to 2.0 ± 0.21 × 10^−3^ Bq/m^2^/h with an average value of 1.2 ± 0.11 × 10^−3^ Bq/m^2^/h. The mass and area exhalation rates were determined for the three categories of the study area. Mass exhalation rates for mining areas ranged from 2.8 ± 0.2 to 4.2 ± 0.4 × 10^−5^ Bq/kg/h (mean = 3.6 ± 0.3 × 10^−5^ Bq/kg/h), 3.0 ± 0.3 to 6.5 ± 0.7 × 10^−5^ Bq/kg/h (mean = 4.5 ± 0.5 × 10^−5^ Bq/kg/h) for farming areas and 3.4 ± 0.3 to 5.1 ± 0.5 × 10^−5^ Bq/kg/h (mean = 4.3 ± 0.4 × 10^−5^ Bq/kg/h) for undisturbed areas. Area exhalation rates were also evaluated as 0.8 ± 0.07 to 1.3 ± 0.12 × 10^−3^ Bq/m^2^/h (mean = 1.1 ± 0.09 × 10^−3^ Bq/m^2^/h) for mining areas, 0.9 ± 0.08 to 2.0 ± 0.21 × 10^−3^ Bq/m^2^/h (mean = 1.4 ± 0.12 × 10^−3^ Bq/m^2^/h) for farming areas and 1.0 ± 0.08 to 1.5 ± 0.14 × 10^−3^ Bq/m^2^/h (mean = 1.3 ± 0.11 × 10^−3^ Bq/m^2^/h) for undisturbed areas. From the data presented, mining areas recorded low exhalation rates than observed for farming and undisturbed areas. This could be attributed to the fact that human activities which result in soil disturbance leading to the escape of radon gas from soils are less in farming and undisturbed areas (residential and non-residential) and thus radon gas are trapped in soil pores with less means of escape into the environment [27,28] whereas the low exhalation rates recorded for mining areas can be due to the escape of Rn-222 gas from soils through the continuous mining activities.

The overall highest value of mass and area exhalation rates of and 6.5 ± 0.7 × 10^−5^ Bq/kg/h and 2.0 ± 0.21 × 10^−3^ Bq/m^2^/h, respectively were obtained from a farming area (SL08). These also corresponded to the highest Ra-266 and Rn-222 activity concentrations being recorded at the same location. A linear relationship was observed between Ra-226, Rn-222 and exhalation rates in the investigated soil samples of this study. The mass and area exhalation rates increased with higher Ra-226 concentration in the soil samples as it was observed that sampling areas which recorded high Ra-226 concentrations had high exhalation rates [2,31]. The high exhalation rates evaluated in some sample locations indicate that there is a radiological effect associated with the use of such soils as building materials [44,54]. The variation in mass and area exhalation rates from one location to the other maybe attributed to the varying Ra-226 activity concentrations and the geological formations of the various sampling locations in the study area [2,57,58]. It can also be attributed to the different soil types, soil particle sizes, soil porosity and surface crystallography [44,59]. Rn-222 gas moves easily through sandy soils which are permeable than clay soils which are impermeable. Therefore, low exhalation rates in an area can be attributed to fine soil particles as low Rn-222 gas from soil means increase in the level of fine particles in the soil. Thus, there is high mobility and emanation of Rn-222 gas in and from soils which are permeable and are of coarse particles [44].

### 3.4 Comparison of results to other studies

The radon exhalation data obtained in this study were compared to those obtained in other regions of the world which used similar measurement method as used in this study. It has been presented in Table 3.

**Table 3:** Comparison between mean Rn-222 activity concentrations and exhalation rates obtained in Atiwa West and those reported in other regions of the world.

Location	Sample type	Radon concentration (Bq/m^3^)	Mass exhalation (E_M_) (mBq/kg/h)	Area exhalation (E_A_) (mBq/m^2^/h)	Reference
Ghana	soil	560.0	0.04	1.2	This study
Palestine	soils	1447.6	–	1459.4	[3]
India	soil	155.5	3.6	14.9	[8]
Lybia	soil	220.3 325.5	8.2 13.9	216.5 320.0	[26]
India	soil	–	4.4	147.1	[31]
Egypt	sand	108.6	70.2	10.8	[32]
India	soil	34.7	1.4	28.3	[35]
India	soil	–	23.1	600.7	[36]
India	soil	61.0	0.02	–	[42]
Pakistan	soil	–	–	375	[43]
Ghana	soil	77.0	–	0.09	[44]
Ghana	soil	61.3 45.4	– –	0.07 0.05	[45]
Iraq	sand	–	22.3	345.9	[60]
Palestine	sand	–	2.4	48.0	[61]
India	sand	–	2.8	93.4	[62]

From Table 3, different Rn-222 activity concentrations and exhalation rates were observed from the various regions. Soil samples from Palestine recorded the highest Rn-222concentration of 1447.6 Bq/m^3^ followed by 560.0 Bq/m^3^ obtained in this study. The least Rn-222 concentration (34.7 Bq/m^3^) was recorded in India. An area in Ghana also had a low Rn-222 of 45.4 Bq/m^3^ as compared to 560.0 Bq/m^3^ in the present study. The difference in these two areas could be attributed to the different geological formations and the anthropogenic activities occurring in the areas [1,4,25,28]. Similarly, soil and sand samples in India showed had varied Rn-222 concentrations though from the same country. The radon area exhalation rates were high in Palestinian and Indian soils. Lybia and Iraq also reported relatively high area exhalation rates. Again, the least area exhalation rate (0.05 mBq/m^2^/h) on the table was reported in Ghana as compared what was observed in the current study. This area had low area exhalation rates due to the low Rn-222 concentrations measured in the soils. In general, low Rn-222 activity concentration resulted in low exhalation and vice versa.

### 3.5 Statistical analyses

#### 3.5.1 Regression analysis of Ra-226, Rn-222 and exhalation rates

**Fig. 6:**
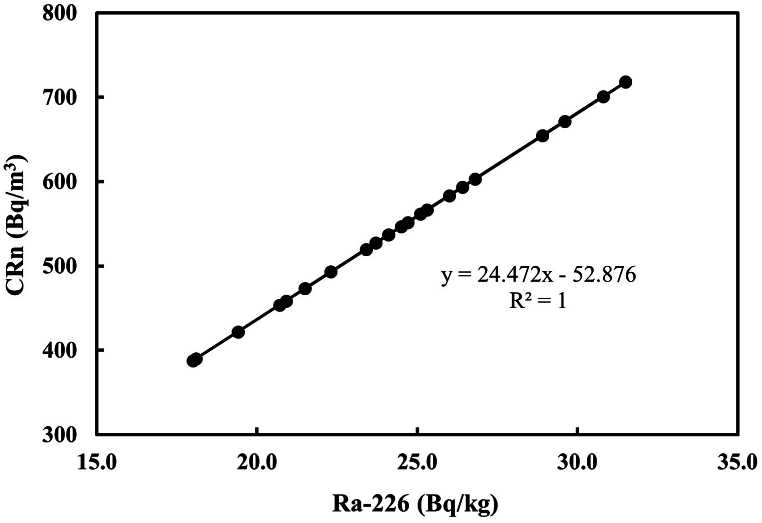
Linear regression of the activity concentrations of Ra-226 and Rn-222.

**Fig. 7:**
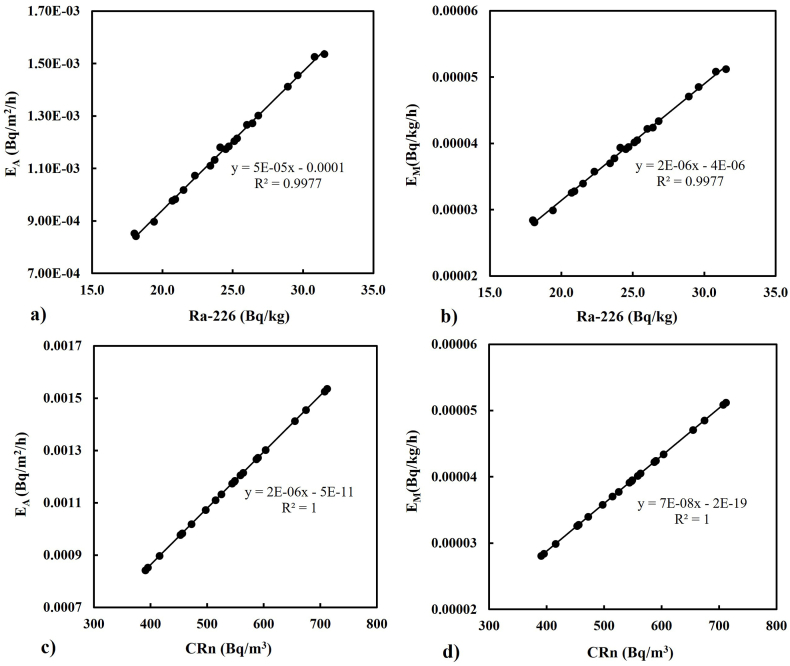
Linear regression between concentrations of Ra-226, Rn-222 and the exhalation rates.

The relationship between Ra-226, Rn-222, and exhalation rates were determined by regression analysis as shown in Fig. 6, Fig. 7. Fig. 6 shows a positive relationship between Ra-226 activity concentration determined by gamma ray spectrometric method and Rn-222 activity concentration (CRn) determined using a passive technique (CR-39 detectors). The R^2^ coefficient was found to be 1. The high R^2^ value proves that Rn-222 concentration measurement is dependent on Ra-226 concentration present in the soil samples. Fig. 7a and b showed that Ra-226 also correlated well with the mass exhalation rates (E_M_) and area exhalation rates (E_A_) having almost the same R^2^ value (0.99) as with Rn-222 indicating that the exhalation rates are determined from the Rn-222 activity concentrations. Rn-222 correlated strongly with the exhalations (R^2^ = 1) as presented in Fig. 7c and d. A good linear relationship was observed between Ra-226, Rn-222 and the exhalation rates. The result of the analysis agrees with similar studies done by other researchers [32,46,[59], [63], [64]].

#### 3.5.2 Pearson's correlation coefficient

The relationship between natural radionuclides, Rn-222 activity concentrations, exhalation rates and radiological hazards were done using Pearson's correlation analysis as shown in Table 4.

**Table 4:** Pearson's correlation coefficient between radionuclides, radon concentrations, exhalation rates and radiological hazards.

	Ra-226	Th-232	K-40	Ra_eq_	H_ex_	H_in_	D	CRn	E_M_	E_A_
**Ra-226**	1									
**Th-232**	0.45	1								
**K-40**	0.34	0.13	1							
**Ra**_**eq**_	0.74	0.90	0.44	1						
**H**_**ex**_	0.74	0.90	0.44	1	1					
**H**_**in**_	0.85	0.83	0.44	0.98	0.98	1				
**D**	0.75	0.88	0.49	0.99	0.99	0.98	1			
**CRn**	0.84	0.16	0.19	0.44	0.44	0.57	0.46	1		
**E**_**M**_	0.84	0.16	0.19	0.44	0.44	0.57	0.46	1	1	
**E**_**A**_	0.84	0.16	0.19	0.44	0.44	0.57	0.46	1	1	1

The analysis showed positive correlation coefficients among the natural radionuclides radiological hazards, and exhalation rates as shown in Table 4. There were weak correlation coefficients observed for Ra-226/Th-232 (0.45) and Ra-226/K-40 (0.34). Ra-226 and Th-232 are from the same parent radionuclide however, in the study area Th-232 exist in abundance with high activity concentrations as compared to Ra-226 hence the relatively coefficient between Ra-226 and Th-232. K-40 and Ra-226 are of different decay series and therefore the reason for the weak correlation between the two radionuclides. Rn-222 (CRn) and Ra-226 showed a strong positive correlation coefficient of 0.84. This demonstrates that Ra-226 is the main source of Rn-222 concentrations in soils since Rn-222 is a daughter product of Ra-226. Therefore Rn-222 emanation from soils is dependent on the levels of Ra-226 and its decay in the environment [44,46,59]. Subsequently, the same coefficient value (0.84) was realized between Ra-226 and the mass and area exhalation rates (E_A_ and E_M_) since their determination is reliant on the Rn-222 concentration. A good correlation was observed between Rn-222 and H_ex_ (0.44) and H_in_ (0.57). It signifies that apart from gamma radiations, radon gas contributes partly to radiation doses and cancer risks associated with the radionuclide in an environment [18,51]. Hence, activities involving use of soils (e.g., building constructions, pottery, brick works) from the study can result in inhalation of radon gas and the risk of having cancer related diseases in the long-term [54].

#### 3.5.3 Cluster analysis

**Fig. 8:**
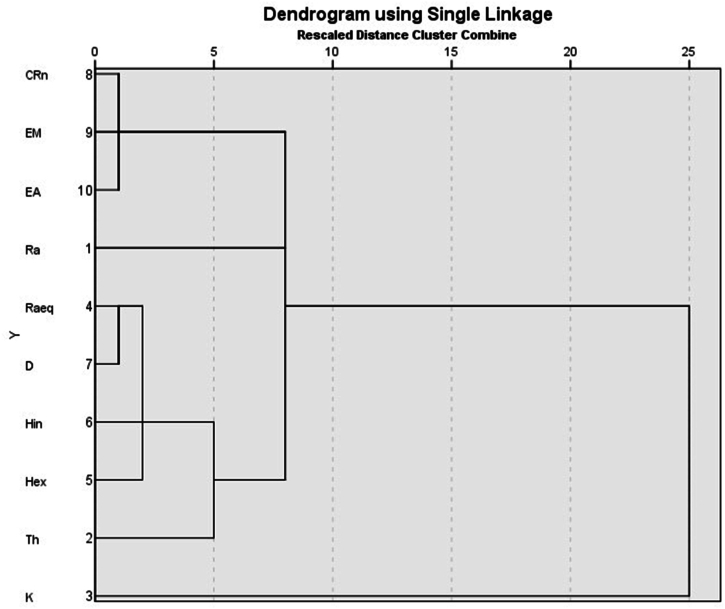
Dendogram of radionuclides, exhalation rates and radiological hazards.

The cluster analysis was done to graphically illustrate the degree of association between the variables. It describes the similarities between natural radionuclides, Rn-222 levels, exhalation rates and radiological parameters. The cluster analysis was performed by applying the single linkage method with correlation coefficient distance between variables. Fig. 8 shows the results of the cluster analysis. According to Fig. 8, three main clusters were observed in the dendogram analysis. The first cluster is made up of CRn, E_M_ and E_A_. The approximately zero distance between CRn, E_A_ and E_M_ variables indicate a 100 % similarity in their measurements and determination [65]. The cluster analysis confirmed that the estimation of E_M_ and E_A_ depends on several factors including the Ra-226 levels in soils. E_M_ and E_A_ rate are due to radon gas levels in soils which is a decay product of Ra-226 [8,29]. Thus, CRn, E_M_ and E_A_ appearing in the same cluster with Ra-226.The second cluster included Ra-226, Th-232, Ra_eq_, H_ex_, H_in_, D. The activity concentrations of Ra-226 and Th-232 constitutes a greater percentage in the determination of Ra_eq_, H_ex_, H_in,_ and D. This means the radionuclides are well correlated with the radiological parameters. The third cluster consisted of K-40. K-40 is not in the same cluster with the other natural radionuclides because it is of a different decay series (K-40 series) as compared to Ra-226 and Th-232 which are of the U-238 decay series though it is of natural source. The results of the cluster analysis agree with the linear regression analysis which showed that Ra-226 well correlated with the exhalation rates.

#### 3.5.4 Principal component analysis (PCA)

**Fig. 9:**
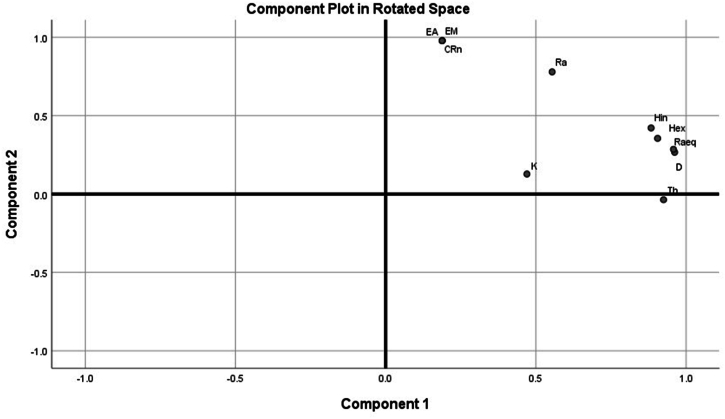
Rotated factor loadings of radionuclides, exhalation rates and radiological hazards.

The PCA explains patterns in variables and displaying the data to emphasize on similarities [51]. It is a statistical tool for assessing the interrelation between variables and describing them with respect to their common factors [66]. In this analysis, the distinctions of variables of the radionuclides, Rn-222 levels, exhalation and radiological hazards were studied. The Kaiser normalization method with varimax rotation was employed to examine the relationship between variables. Only eigenvalues greater than 1 were considered for analysis. Fig. 9 represents the results of the factor loadings. The results of the PCA showed that there were two factors with eigenvalues greater than 1 which explained for 88.81 % of the total variance obtained in the rotated component matrix. According to Table 5, component 1 explained 67.26 % of the total variance loading heavily on Ra-226 and Th-232 and the radiological hazards (Ra_eq_, H_ex_, H_in_ and D) demonstrating that Ra-226 and Th-232 are the main sources of radioactivity levels in the sampled soils and main contributor to the radiation doses. Component 2 accounted for 21.55 % of the total variance. It loaded more on radon concentration and the exhalation rates (CRn, E_M_ and E_A_) and less on Ra-226. This showed relationship between radon levels and their exhalation rates in the sampled soils and thus their concentrations depend on the Ra-226 levels in soils. The results of the PCA are comparable to that of the Pearson correlation and cluster analyses.

**Table 5:** Rotated component matrix.

	Component 1	Component 2
Ra	0.920	0.260
Th	0.698	−0.608
K	0.446	−0.195
Ra_eq_	0.916	−0.395
H_ex_	0.928	−0.291
H_in_	0.952	−0.225
D	0.925	−0.387
CRn	0.795	0.644
E_M_	0.795	0.644
E_A_	0.795	0.644
**Variance explained (%)**	**67.257**	**21.551**

The PCA, Pearson correlation, cluster and regression analyses explained the interrelationships between the natural radionuclides, radiological hazards, Rn-222 concentrations and the exhalation rates determined in soil samples from the study area.

### 3.6 Assessment of radiological risks

The estimated radiation doses and hazards from soil samples in this study are presented in Table 6.

**Table 6:** Radiological hazards of soil samples from the various sampling locations.

Location	Ra_eq_	H_ex_	H_in_	D (nGy/h)
SL01	130.7	0.4	0.4	58.5
SL02	146.3	0.4	0.4	64.6
SL03	139.4	0.4	0.4	60.6
SL04	82.1	0.2	0.3	37.4
SL05	114.9	0.3	0.4	52.5
SL06	112.9	0.3	0.4	49.8
SL07	87.6	0.2	0.3	39.3
SL08	261.7	0.7	0.9	117.7
SL09	103.2	0.3	0.3	47.7
SL10	159.5	0.4	0.5	71.1
SL11	162.0	0.4	0.5	73.1
SL12	124.7	0.3	0.4	54.5
SL13	128.0	0.3	0.4	55.8
SL14	118.0	0.3	0.4	52.8
SL15	161.6	0.4	0.5	72.9
SL16	126.2	0.3	0.4	55.1
SL17	108.8	0.3	0.4	49.3
SL18	97.9	0.3	0.3	46.1
SL19	107.3	0.3	0.4	48.9
SL20	121.2	0.3	0.4	54.1
SL21	116.6	0.3	0.4	52.3
SL22	94.6	0.3	0.3	42.2
**Average**	**127.5**	**0.3**	**0.4**	**57.1**
**World**	**370.0**	**1.0**	**1.0**	**60.0**

The radium equivalent (Ra_eq_), which compares the specific activities of radionuclides in a sample is assumed to produce the same gamma dose [67]. The Ra_eq_ ranged from 82.1 to 261.7 Bq/kg. The mean Raeq was found to be 127.5 Bq/kg which is below the reference level of 370 Bq/kg corresponding to an effective dose of 1 mSv. Thus, the population of the area is likely to receive radiation dose below the maximum permissible level of 1 mSv due to the low Ra_eq_ values [54,68]. The external and internal hazard indices (H_ex_ and H_in_) evaluate the hazards of natural gamma radiation on human health. In order to keep radiation hazard insignificant, the indices must be less than unity. The H_ex_ values obtained in this study ranges from 0.2 to 0.7 with a mean of 0.3 while the H_in_ values were from 0.3 to 0.9 with a mean of 0.4. H_ex_ and H_in_ having values less than unity means the natural gamma radiation in soils from the study area will not cause respiratory complications such as asthma and cancer [55,67]. The absorbed gamma dose rate (D) in air 1 m above the ground were found to range from 37.4 to 117.7 nGy/h. The mean value of 57.1 nGy/h was beneath the reference value of 60.0 nGy/h (UNSCEAR, 2000). However, some locations (27 %) recorded gamma dose rates above than the reference value especially in locations with very high Th-232 activity concentrations.

## 4 Conclusion

The activity concentration of Ra-226, Th-232 and K-40 in soils, radon activity concentration, mass and area exhalation rates have been studied in soils from Atiwa West district of the Eastern region of Ghana. The associated radiological risks and the estimation of lung cancer cases per a million people were also evaluated. Ra-226 concentrations were below worldwide averages while Th-232 were above and consequently contributed significantly to the estimated radiation hazards. The study showed that Rn-222 concentrations ranged from 390.6 ± 38 to 907.5 ± 93 Bq/m^3^ with an average value of 560.0 ± 54 Bq/m^3^. The mean mass and area exhalation rates were evaluated to be 4.0 ± 0.4 × 10^−5^ Bq/kg/h and 1.2 ± 0.11 × 10^−3^ Bq/m^2^/h, respectively. The highest mass and area exhalation rates were recorded in a farming area. Therefore, it signifies that there is a degree of radiological effect associated with the use of such soils as building materials by the public. The assessed radiation hazard parameters were below recommended guidelines. Statistical analyses performed on the obtained data explained the relationship between the natural radionuclides, radon concentrations, exhalation rates and radiological indices estimated in soils samples from the study area.

## Data availability statement

All data presented in the article.

## CRediT authorship contribution statement

**Esther Osei Akuo-ko:** Writing – original draft, Investigation, Data curation. **Francis Otoo:** Writing – review & editing, Writing – original draft, Conceptualization. **Eric Tetteh Glover:** Methodology, Investigation, Data curation. **Eunice Amponsem:** Visualization, Validation, Project administration, Investigation. **Amin Shahrokhi:** Project administration, Formal analysis, Data curation, Conceptualization. **Anita Csordás:** Writing – original draft, Supervision, Investigation, Formal analysis. **Tibor Kovács:** Writing – review & editing, Writing – original draft, Supervision, Funding acquisition, Conceptualization.

## Declaration of competing interest

The authors declare that they have no known competing financial interests or personal relationships that could have appeared to influence the work reported in this paper.
